# Dietary Implications of Detoxified *Jatropha curcas* Kernel for *Clarias gariepinus* Fingerlings

**DOI:** 10.3390/vetsci8080152

**Published:** 2021-07-30

**Authors:** Victor Tosin Okomoda, Sarah Ojonogecha Musa, Lateef Oloyede Tiamiyu, Shola Gabriel Solomon, Cosmas Chidiebere Alamanjo, Ambok Bolong Abol-Munafi

**Affiliations:** 1 Department of Fisheries and Aquaculture, College of Forestry and Fisheries, Federal University of Agriculture Makurdi, Makurdi PMB 2373, Nigeria; solagabriel@yahoo.co.uk; 2 Higher Institution Centre of Excellence (HICoE), Institute of Tropical Aquaculture and Fisheries (AKUATROP), Universiti Malaysia Terengganu, Kuala Nerus 21030, Malaysia; 3 Department of Zoology, Faculty of Natural Sciences, University of Jos, Jos PMB 2084, Nigeria; musasom@yahoo.com; 4 Department of Aquaculture and Fisheries, Faculty of Agriculture, University of Ilorin, Ilorin PMB 1515, Nigeria; lottiamiyu@yahoo.com; 5 Department of Agricultural Technology, Federal College of Forestry, Jos PMB 2019, Nigeria; cosmas.alamanjo@fcfjos.edu.ng; 6 Faculty of Food Science and Fisheries, Universiti Malaysia Terengganu, Kuala Nerus 21030, Malaysia

**Keywords:** antinutritional components, soaking, African catfish, jatropha kernel, non-conventional feedstuff, feed processing

## Abstract

Antinutritional components must be substantially reduced to ensure better utilization of unconventional feeds in animal nutrition. Among the different methods of processing, soaking represents a simple and inexpensive alternative. This study attempted to determine the nutritional properties of socked *Jatropha curcas* kernel (JCK) and the effect of its dietary inclusions on *Clarias gariepinus* (*n* = 50; mean initial weight = 6.19 ± 0.52 g). Three treatments of JCK (i.e., soaking 24, 48, and 72 h) were tested alongside a control group. The result showed that soaking substantially improved proximate composition and reduced anti-nutrient compared to the control JCK. A similar trend of improved performance was observed when the treated JCK was included in diets composed of 35% crude protein; a total of 315 kcal g^−1^ energy and fed to *C. gariepinus* for 56 days. Alongside the improvement observed in growth, it was also noted that carcass protein and haematological variables were improved with the dietary administration of soaked JCK. Histological examination of the intestine and liver tissues also revealed fewer signs of histopathological degeneration in the fish, consequent upon dietary inclusion of soaked JCK (i.e., 72 h) compared to those raised on the raw JCK-included diets. It was, therefore, concluded that soaking could be a much easier method of nutritionally improving JCK for the administration to *C. gariepinus.*

## 1. Introduction

The African catfish, *Clarias gariepinus*, is prided as being among the most economically important food fish popularly cultured in Africa, Asia, Europe, and Latin America [1,2]. The successful propagation of the African catfish around the world can be linked to certain qualities of the fish, which includes its high fecundity, growth performance, tolerance to environmental stresses, and a relatively attractive price per kilogram [3,4]. Although *C. gariepinus* has an omnivorous feeding habit, better growth performance under captivity has been linked to the feeding of a high-quality protein diet largely due to the cannibalistic nature of the fish [5]. Hence, to meet the nutritional requirement of the fish, expensive feed ingredients such as fishmeal is used, consequently, increasing the feeding and fish production cost [6,7,8,9]. Therefore, to ensure a productive catfish venture, there is a need to identify alternative quality feed ingredients that can be used in place of expensive fishmeal without compromising the optimum growth of the fish [10].

There are many such potential alternative protein ingredients and may include various types of animal/animal by-products and different terrestrial plant meals widely distributed around the world [11]. Although the importance of animal protein alternatives has been demonstrated in many previous studies [12,13], research has been intensified on plant protein sources because of their low environmental impacts and for economic reasons [14]. Soybean meal is one of the most researched plant protein alternatives [11]. However, its dominance as a viable alternative to fishmeal (due to its high nutritive composition such as quality protein and amino acids), has caused overdependence from many competing interest groups [15]. Consequently, a higher demand for the soybean meal has caused a hike in prices to a point where inclusions at a high level in animal feed seem no longer economically viable [16]. This, therefore, has attracted attention to the use of non-conventional feed resources (NFRs) which are cheaper and unused by humans for nutritional purposes.

In the search for suitable NFRs, much attention should be given to identifying a candidate that can support the growth of the animal without deleterious effect on the fish’s health [8,9,17,18]. Unfortunately, some research have shown that the dietary incorporation of alternative protein ingredients of plant origin for fish would cause poor performance and the display of several histopathological degenerations [19]. This could be linked to the nutritional inferiority of the selected NFRs compared to conventional feedstuffs [20] or the inability of the fish to effectively utilize the nutrients in the NFRs because of the presence of antinutrient [8]. The utilization of NFRs in the latter category can be improved by various processing methods [21]. While some processing methods can increase the cost of feed production (due to the extra energy needed for thermal and mechanical processing), soaking is one of the few low-cost and simplistic methods that can be used for the processing of NFRs [22,23,24,25]. However, compared to other methods, this appears to be the least researched for the detoxification of many NFRs, perhaps because they are thought to be ineffective against thermolabile antinutrients.

*Jatropha curcas* is one of the NFRs that is starting to gain prominence in animal research. This is partly because it has high crude protein, lipids, and a well-balanced amino acid profile which compares well with fishmeal and soybean [8,9]. *J. curcas* is a member of the *Euphorbiaceae* family and its economic importance has been demonstrated in its exploitation for several purposes [26]. However, the major drawback to the commercial application of this widely distributed NFR in animal nutrition is the abundance of antinutrients which includes, but is not limited to, phytate, trypsin inhibitor, lectin, etc. [27]. Although attempts have been made to detoxify and improve the by-product of *J. curcas*, i.e., the kernel (JCK), as a protein supplement in the diet of various fish [28,29,30], no study has evaluated the dietary implication of soaking the JCK in fish nutrition. This study, therefore, was designed to evaluate the proximate component, amino acid profile and antinutrients of soaked *Jatropha curcas* kernel (JCK) as well as to determine the effect of their dietary inclusions in the diet of African catfish *C. gariepinus* fingerlings.

## 2. Materials and Methods

### 2.1. Feedstuff Procurement and Processing

JCK was obtained similar to the process described in our earlier reported studies [8,9]. Briefly, the Jatropha fruits were obtained from farmlands located at the Ofoke—Ojope, Apa L.G.A in Benue State, Nigeria. They were then sundried and crushed to remove the seed from its husks. Thereafter, the JCK was obtained by crushing the seeds. The JCK obtained in bulk was then shared into four batches; one set was used as the control treatment (i.e., raw JCK without processing), while the other three batches were soaked in iron buckets filled with distilled water for 24, 48, and 72 h. This was conducted in three replicates. The room condition where the soaking of JCK was conducted was maintained at 36 °C with 65 ± 0.56% relative humidity. Thereafter, the various treatment replicates were dried in an oven to constant weight for 3 h (drying temperature = 60 °C) before sub-samples were nutritionally analysed.

### 2.2. Feedstuff Nutritional Analysis and Diet Formation

The proximate composition and antinutritional factors (ANFs) of the JCK were conducted at the Department of Zoology laboratory, University of Jos, Nigeria. Protein, moisture, lipid, and ash content were obtained through the method by the AOAC (Association of Official Analytical Chemists) [31], while the nitrogen-free extract was obtained by difference (i.e., the sum of other ingredients minus from 100%). Antinutrients such as phytic acid [32], total oxalate [33], cyanogenic glycosides [34], trypsin inhibitor activity [35], and phytate [36] were also evaluated (as shown in Table 1). Upon obtaining the nutritional components of the unconventional feed ingredients, they were milled separately by their different treatments and stored until the experimental diets were formulated. For the diet formulation, other feed ingredients were used, namely, soybeans meal (seeds toasted using the methods previously described by Okomoda et al. [9] and Tiamiyu and Solomon [37] before milling), fish meal, cassava flour, vitamin/mineral premixes, rice bran, and maize meal. Hence, four diets composed of 35% crude protein (CP) and 315 kcal g^−1^ energy were obtained using the combination of the feed ingredients as shown in Table 2. Following the thorough mixing of the feed ingredients (to obtain a uniform meal), a dough was formed by the addition of warm water (60 °C) and continuous stirring. The dough was then pelleted through a 2 mm die, sundried, and packaged until it was needed to feed the experimental fish.

### 2.3. Zootechnical Evaluation of Fish Performance

The two hundred fingerlings of the African catfish used for this study were of the same breeding history and obtained from Miracle farm situated at Jos, Plateau State in Nigeria. Since the same farm facility was used for the research, they were not acclimatized before the start of the study. Fifty progenies of the *C. gariepinus* weighing 6.19 ± 0.52 g were distributed in triplicate to twelve concrete tanks measuring 2 × 1 × 1 m^3^ (stocking density of 5 gL^−1^). The tanks were fitted with taps connected to the reservoir to maintain a continuous flow-through aquaculture system throughout the study period. This kept the water quality of the different treatment ponds at optimum (T °C = 27.1 ± 0.31 °C; pH = 7.03 ± 0.2; Cond. = 652 ± 0.11 µS/cm; TDS = 210 ± 0.23 mg L^−1^; Dissolved Oxygen = 5.23 ± 0.11 mg L^−1^). The monitoring of the water quality was achieved using Hanna’s digital multi-parameter water checker (Model HL 98126). The feeding of the fish in each treatment group was based on 5% of their body weight daily. The daily rations were sub-divided into two parts for the morning (8 a.m.) and evening (6 p.m.) administration. The remnants of the feed after each feeding was siphoned from the rearing tanks, sundried and weighed to determine the actual feed intake by the fish.

To obtain the zootechnical parameters of the fish, they were weighed in bulk biweekly with the aid of a sensitive weighing balance (approximately 0.001 g sensitivity), and the number of surviving fish determined to estimate mortality during the experimental period. Consequently, both bulk weight and mortality were used to adjust the feed administered daily to the fish until the next rounds of measurements were performed. After 56 days of the feeding trials, the growth performance indices of the experimental fish were determined with the relations shown below:

(a) Growth rate (g/d) = $\frac{W_{2}-W_{1}}{t_{2-t_{1}}}$; where W_1_ = initial weight (g); W_2_ = final weight (g); t_2_ − t_1_ = duration between W_2_ and W_1_ (days)
(b) Specific growth rate (%/day) = $\frac{\log_{e}\left(W_{2}\right)-\log_{e}\left(W_{1}\right)}{t_{2-t_{1}}}\times 100$;
(c) Feed conversion ratio (FCR) = $\frac{\mathrm{dry}\;\mathrm{feed}\;\mathrm{intake}}{W_{2}-W_{1}}$;
(d) %Survival = $\frac{\mathrm{fish}\;\mathrm{stocked}-\mathrm{mortality}}{\mathrm{fish}\;\mathrm{stocked}}\times 100$.

Nutritional composition (proximate analysis) of the carcass of the different fed fish, as well as the diet fed, was determined in triplicate at the end of the study using the method described by the AOAC [31].

### 2.4. Blood Analysis of the Experimental Fish

Similar to the process used in our earlier studies on JCK [8,9], blood samples of fish in the different treatments were collected at the end of the feeding trial from three to four anesthetized fish (according to the methods of Klontz and Smith, [38]). The fish were anesthetized using 100 mg/1 solutions of tricaine methanesulphonate (MS222). The blood was then obtained from the caudal peduncle using a needle fitted on a sterilized syringe. This was, thereafter, discharged into 1.5 mL heparinized plastic tubes and placed on ice. The haematocrit (HCT) (also known as packed cell volume (PCV)) value was determined by microcentrifugation of the blood in heparinized microhaematocrit tubes [39,40]. Haemoglobin concentration (Hb) was obtained using the cyanmethemoglobin procedure [41,42]. The red blood cell value (RBC) value, however, was taken following microscopic observation with the Neubauer haemocytometer coulter-model T 540 [43]. The other parameters, i.e., the mean corpuscular volume (MCV), mean corpuscular haemoglobin (MCH), and mean corpuscular haemoglobin concentration (MCHC) were determined by calculation using the relation given by Klinger et al. [44]:

(1) $$\mathrm{MCV}=\frac{\mathrm{PCV}\times 1000}{\mathrm{RBC}\times 10^{12}}$$

where PCV = Packed Cell Volume, RBC = Red Blood Cell Count

(2) $$\mathrm{MCH}=\frac{\mathrm{Hb}\left(g.L^{-1}\right)}{\mathrm{RBC}\times 10^{12}.L^{-1}}$$

where Hb = Haemoglobin Concentration, RBC = Red Blood Cell Count

(3) $$\mathrm{MCHC}=\frac{\mathrm{Hb}\left(g.L^{-1}\right)}{\mathrm{PCV}\;\left(L.L^{-1}\right)}$$

### 2.5. Histological Analysis of Tissues

Intestine and liver tissues of the experimental fish (i.e., those fed control and JCK soaked for 72 h) were fixed in 10% saline for forty-eight hours [9]. Thereafter, they were processed (i.e., dehydrated, diaphanized and infiltrated) using the automatic duplex processor, Standon, and Southern (Model: C. 35H). The processed tissue were then embedment in paraffin wax. Micro-sectioning of the tissue (i.e., at 5 µm) [45] was conducted before placing on slides. They were then dewaxed and dehydrated before staining in Harris’s haematoxylin and eosin (H&E). The processed slides were fixed with Canada balsam and the tissue subsequently observed with the Nikon Eclipse 80i compound microscope.

### 2.6. Data Analysis

Minitab 14 for Windows (Minitab Inc., State College, PA, USA) was used to perform the data analysis in the current study. The summary statistics of the different variables measured across the treatment and control groups were followed by a test of normality and homogeneity of variance. Thereafter, the analysis of variance (ANOVA) and separation of means through the Fisher’s least significant difference (*p* ≤ 0.05) were performed. Since the assumption for normality and homogeneity was upheld, there was no need to use the Kruskal–Wallis non-parametric test.

## 3. Results

The proximate analysis of the JCK showed that prolonged soaking increased protein but reduced fat and ash significantly compared to the raw JCK (Table 1). Additionally, the ANFs isolated, namely, phytic acid, oxalate, cyanogenic glycoside, phytate, and trypsin inhibitor, consistently reduced with the prolonged soaking of the JCK. Using the processed/raw JCK combined with other ingredients, four experimental diets obtained were observed to contain about 35% protein and 315 kcal g^−1^ in gross energy. The gross and proximate compositions of the formulated diet are as presented in Table 2.

The outcome of feeding the experimental diet on the growth of *C. gariepinus* at the end of 56 days is as shown in Table 3. The inclusion of soaked JCK seemed to promote the dietary utilization of the JCK by *C. gariepinus*. This was demonstrated by the observation of a higher body weight gained as well as a lower FCR in the treatment groups when compared to those fed raw inclusions of JCK. Additionally, the protein was accumulated much more in the treatment groups compared to the control as revealed by the proximate composition of the experimental fish carcass. However, fat content was reduced in the treatment groups (Table 4).

Haematological parameters were as revealed in Table 5. Our findings show higher values of HCT, Hb, and RBC with inclusions of soaked JCK compared to those fed a diet including the raw JCK. However, similar values of MCV, MCH, and MCHC were obtained in both treatment and control groups.

**Figure 1:**
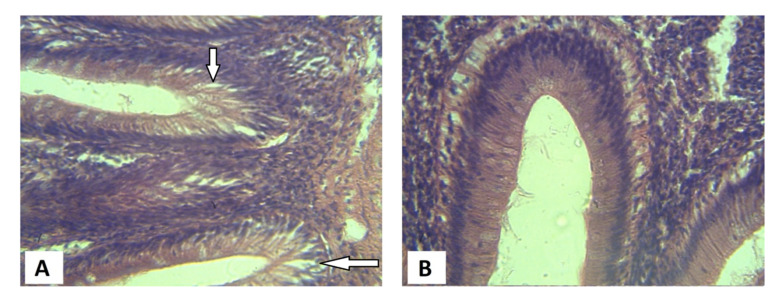
Microsection of the intestine of African catfish *C. gariepinus* fed inclusion of (**A**) raw *J. curcas* kernel; (**B**) and 72 h socked *J. curcas* kernel (arrows shows sloughed epithelial linings).

**Figure 2:**
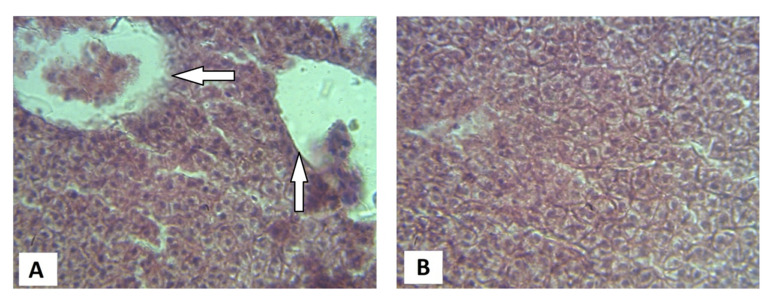
Microsection of the Liver of African catfish *C. gariepinus* fed inclusion of (**A**) raw *J. curcas* kernel; (**B**) and 72 h socked *J. curcas* kernel (arrows show portions of sloughed/necrotic liver cells).

Histological examination of the intestine of the *C. gariepinus* fed the dietary inclusion of the raw JCK showed the depletion of the goblet cells and the sloughing-off of the epithelial lining (Figure 1). However, in the treatment group, there was an observed increased proliferation of goblet cells while the epithelial lining appeared intact. The liver of the *C. gariepinus* fed raw JCK showed high levels of necrosis; however, the necrotic cells seem to reduce in the fish fed soaked JCK (Figure 2).

## 4. Discussion

Soaking of JCK, as seen in this study, appears to be an effective method for improving the nutritional composition of the feed ingredient. This is because of the duration-dependent increase in the crude protein of JCK observed with soaking. This is similar to the observation reported by Johnson et al. [30] for *J. curcas* seed soaked for up to seven days. The report of Kajihausa et al. [24] and Solomon et al. [25] also found that soaking was effective in improving the nutritional composition of sprouted sesame seed flour and pigeon pea *Cajanus cajan*, respectively. Fat and ash were, however, observed to be reduced in the soaked JCK and were thought to have been leached in the water which was subsequently drained off. The improvement in protein, therefore, may be explained by a condensation made possible through the leaching of minerals and fat as well as the dehydration process during the oven drying of the JCK. Soaking also substantially reduced all of the antinutrients isolated in the current study. The findings of Nwosu [22] on asparagus beans (*Vigna sesquipedalis*) had earlier shown that soaking could effectively reduce phytate and cyanogenic glycosides. Additionally, Makinde et al. [23] reported an oxalate reduction of between 21.69% and 29.64% when different parts of *Sesamum indicum* cultivars were soaked for 12 h.

Prolonged duration-based efficiency in the reduction in antinutrients such as cyanogenic glycosides had also been demonstrated in the study by Rawat et al. [46], with the shoots of bamboo *Dendrocalamus giganteus* and *D. hamiltonii* soaked between 12 and 24 h. Additionally, the study reported by Abou-Arab and Abu-Salem [47] showed that soaking significantly decreased the phytic acid content and trypsin inhibiting activity of *J. curcas*-defatted whole seed and kernel seeds. Although, the action of water in reducing the antinutrient may not be fully understood, however, it is assumed that possible leaching of antinutrient ions may have occurred through the osmotic action of water. This may be verified in future studies by determining the phytochemical composition of the wastewater discarded after soaking the JCK. No doubt, soaking substantially reduced several antinutrients in this study, however, this processing method is not as effective as thermal processes earlier reported by Musa et al. [8] and Okomoda et al. [9] for the same strain and species of the JCK. The study by Abou-Arab and Abu-Salem [47] had earlier justified this position when they tested different processing methods on *J. curcas* seeds and kernels. Nevertheless, this seemed not to have a deleterious effect on the zootechnical parameters of the fish as observed in the current study.

Several factors were hypothesized to contribute to the effective utilization of *J. curcas* in the diet of fish; among which is the level of toxic antinutrients [40]. The better performance with the feeding of treatment groups could imply that the antinutrients in the soaked JCK were below the level that could cause harm to the fish. Previous nutritional studies on variously detoxified JCK in the diet of Nile tilapia [48], Carp [28], and African catfish *C. gariepinus* [9] have reported FCR ranges below two. The observation of an FCR of less than 1.5 in the current study was evident that despite the simplistic and inexpensive nature of the processing method adopted in the current study, it was effective for JCK detoxification, hence, improving its utilization by *C. gariepinus*. Another possible advantage of this method of processing is that prolonged duration seems to enhance the feed without having a shorter optimum duration (at least to the 3rd day of soaking). The finding reported by Okomoda et al. [9] with JCK co-treated using 62 min hydrothermal treatment alongside prolonged fermentation of up to 21 days, showed that this was not also detrimental to the nutrients in the feed ingredient nor the fish fed with it. The hydrothermal treatment used in Okomoda et al.’s [9] study had been earlier optimized by Musa et al. [8], where it was observed that beyond 62 min of thermal processing, vital nutrients of the JCK were denatured; consequently, affecting the zootechnical parameters of the fish fed with it.

Not only was the growth better, but carcass protein was also higher with inclusions of JCK soaked for prolonged durations. A similar increase in body protein was earlier reported in *Cyprinus carpio* fed a detoxified Jatropha-incorporated diet [49]. According to Jahan et al. [50], a higher protein content in fish is a pointer to the fact that the administered diet has the proper and balanced amino acids to support muscle growth. Hence, the prolonged soaking of JCK in this study may be stated to have improved nutrient accumulation of the fish. However, lipid contents of the carcass fed the soaked JCK appeared to be inversely related to protein. This negates the trend reported in the study by Hasan et al. [51] on common carp, where better lipid storage in the carcass was consequent upon the inclusions of protein supplements of plant origin as a substitute for fishmeal. Lipid content in carcass has been stated to be a function of lipid deposition in the liver [52]. Therefore, the lower lipid in the treatment group is suggestive that the better growth observed was likely a function of protein accumulation in the fish rather than lipid. This is similar to our earlier findings on *C. gariepinus* fed JCK hydrothermally processed [8] and those co-processed with fermentation [9].

Among the antinutrient in *J. curcas*, the phytic acid molecules are capable of forming complexes with iron or the amine group. This action, therefore, reduces the availability of the same for the biosynthesis of biologically important cells as well as increasing erythrocyte fragility [53]. It is, therefore, not surprising to observed higher Hb, RBC, and HCT in the fish groups fed soaked JCK compared to that fed the control diet. To achieve a similar higher Hb and RBC in the study by Kumar et al. [49], the authors had to include phytase in the diet in order to degrade the excessive phytic acid levels in the Jatropha-based diet fed to the carp. Although plant ingredients have been thought to increase the early release of immature erythrocytes [54], our finding, however, suggests that the antinutrient composition of the plant ingredient may be a determinate factor in the level of erythrocyte release. Since Hb and Hct values have been frequently used as indicators of the wellbeing and health status of fish [55], it could be rightly stated then that soaking JCK and administering the same in the diet of African catfish improved the health status of the fish.

In the same vein, the liver and intestinal tissue had lesser histopathological symptoms for fish fed the soaked JCK compared to those fed the raw JCK. One of the observable indicators of intestinal abnormality caused by infections or nutritional imbalances is the depletion of the goblet cells [56]. This was largely evident in the control group in the current study. The finding by Kumar et al. [49] had also opined that an excessive antinutrient in Jatropha (such as those reported in the raw control of the current study) causes significant liver damage. Additionally, Kumar et al. [28] had reiterated in their study that the necrosis of enterocytes on the tips of the intestinal villi of fish fed a Jatropha-based diet affected digestion and absorption of nutrients. Hence, the histological results observed may help explain the perceived decrease in nutrient utilization observed in the African catfish administered the control diet as compared to those given the treatment diet.

## 5. Conclusions

It is concluded, that soaking of JCK can significantly improve the feed ingredient and their utilization by African catfish *C. gariepinus*. Although performance was improved with prolonged soaking in this study, some antinutrients are known to be thermolabile. Hence, co-treatment of soaked JCK with other thermal processing methods could be the focus of future research to further improve their utilization in the diet of fish. Studies on the substitution of conventional feed ingredients (such as fishmeal and soybean meal) can also be attempted in the future to completely replace these expensive feed ingredients in the diet of fish.

## Figures and Tables

**Table 1:** Proximate analysis and antinutrients of *Jatropha curcas* kernel socked for different durations (g 100 g^−1^). Numbers are means ± standard errors.

Parameter	Soaking Time			
	0 h	24 h	48 h	72 h
Proximate composition (%)				
Crude Protein	55.29 ± 0.39 ^c^	56.10 ± 0.11 ^b^	56.19 ± 0.30 ^b^	57.02 ± 0.12 ^a^
Fat	9.45 ± 0.50 ^a^	9.32 ± 0.14 ^a^	8.72 ± 0.11 ^b^	8.69 ± 0.26 ^b^
Ash	8.91 ± 0.12 ^a^	8.50 ± 0.10 ^b^	8.25 ± 0.31 ^c^	8.17 ± 0.38 ^d^
Crude Fibre	8.59 ± 0.22 ^c^	8.75 ± 0.02 ^c^	9.22 ± 0.03 ^b^	9.73 ± 0.05 ^a^
Moisture	1.79 ± 0.02 ^d^	2.19 ± 0.11 ^c^	2.69 ± 0.01 ^b^	2.98 ± 0.03 ^a^
NFE	15.96 ± 0.55 ^a^	15.14 ± 0.14 ^b^	14.92 ± 0.19 ^b^	13.39 ± 0.13 ^c^
Antinutrients (g 100 g^−1^)				
Phytic Acid	12.29 ± 0.42 ^a^	9.50 ± 0.16 ^b^	7.55 ± 0.11 ^c^	5.82 ± 0.02 ^d^
Oxalate	1.15 ± 0.11 ^a^	0.93 ± 0.04 ^b^	0.48 ± 0.02 ^c^	0.41 ± 0.00 ^d^
Cyanogenic Glycoside	0.83 ± 0.34 ^a^	0.60 ± 0.03 ^b^	0.43 ± 0.01 ^c^	0.23 ± 0.02 ^d^
Phytate	3.49 ± 0.39 ^a^	0.28 ± 0.03 ^b^	0.03 ± 0.01 ^c^	0.04 ± 0.01 ^c^
Trypsin Inhibitor	2.99 ± 0.13 ^a^	2.18 ± 0.08 ^b^	2.14 ± 0.06 ^b^	1.91 ± 0.02 ^c^

NFE—nitrogen-free extract. Means in the same row with different superscripts differ significantly (*p* ≤ 0.05).

**Table 2:** Gross and proximate composition (%) of the diets included with soaked *Jatropha curcas* kernel at different durations. Numbers are means ± standard errors (gross composition = feed amount per kg of diet formulated).

Parameter	Soaking Time			
	0 h	24 h	48 h	72 h
Diet formulation (g k^−1^)				
*Jatropha curcas*	294.80	294.90	291.80	291.20
Soybean	413.20	410.70	459.90	462.20
Yellow maize	65.80	66.90	45.60	42.50
Cassava flour	15.80	16.10	11.00	10.20
Rice bran	60.30	61.40	41.80	38.90
Fish meal	100.00	100.00	100.00	100.00
* Vit/Min premix	50.00	50.00	50.00	50.00
Proximate analysis (%)				
Crude Protein	35.41 ± 0.12 ^a^	35.85 ± 0.12 ^a^	35.58 ± 0.16 ^a^	35.12 ± 0.15 ^a^
Fat	7.16 ± 0.36 ^b^	6.76 ± 0.32 ^c^	7.12 ± 0.20 ^b^	7.37 ± 0.25 ^a^
Ash	8.22 ± 0.23 ^a^	5.89 ± 0.42 ^b^	5.54 ± 0.21 ^c^	5.33 ± 0.17 ^c^
Crude fibre	6.89 ± 0.32 ^a^	5.59 ± 0.15 ^b^	5.74 ± 0.23 ^b^	4.16 ± 0.11 ^c^
NFE	27.43 ± 0.39	27.09 ± 0.17	27.26 ± 0.54	27.34 ± 0.95
Energy (kcal g ^−1^)	314.91 ± 1.34	314.83 ± 1.02	315.01 ± 2.00	315.21 ± 0.4

* Vitamin premix and mineral premix are contained in Okomoda et al. [9]. Nitrogen-free extract (NFE): 100—(the sum of other nutrients). Means in the same row with different superscript differ significantly (*p* ≤ 0.05)

**Table 3:** Zootechnical parameters of *Clarias gariepinus* fed soaked *Jatropha curcas* kernel-based diets. Numbers are means ± standard errors.

Parameter	Soaking Time			
	0 h	24 h	48 h	72 h
Final weight (g)	9.95 ± 0.05 ^d^	14.85 ± 0.05 ^c^	14.60 ± 0.10 ^b^	15.80 ± 0.10 ^a^
Weight gain (g)	3.76 ± 0.15 ^d^	8.66 ± 0.20 ^bc^	8.41 ± 0.10 ^b^	9.61 ± 0.35 ^a^
Specific Growth Rate (g day^−1^)	0.86 ± 0.05 ^c^	1.57 ± 0.06 ^b^	1.54 ± 0.02 ^b^	1.68 ± 0.10 ^a^
Feed Conversion Ratio	3.16 ± 0.14 ^a^	1.40 ± 1.75 ^b^	1.46 ± 0.45 ^b^	1.20 ± 0.56 ^c^
Survival (%)	62.50 ± 0.34 ^d^	86.67 ± 0.58 ^c^	90.00 ± 1.00 ^b^	93.33 ± 0.58 ^a^

Means in the same row with different superscripts differ significantly (*p* ≤ 0.05).

**Table 4:** Carcass proximate analysis (%) of African catfish *Clarias gariepinus* fed soaked *Jatropha curcas* kernel-based diets.

Parameter	Soaking Time			
	0 h	24 h	48 h	72 h
Moisture	78.63 ± 0.56 ^a^	78.26 ± 0.14 ^a^	78.20 ± 0.26 ^a^	78.56 ± 0.24 ^a^
Crude Protein	12.90 ± 0.12 ^c^	13.22 ± 0.73 ^b^	13.20 ± 0.38 ^b^	13.64 ± 0.43 ^a^
Fat	3.69 ± 0.11 ^a^	3.96 ± 0.03 ^a^	3.26 ± 0.06 ^b^	3.07 ± 0.12 ^b^
Ash	2.12 ± 0.13 ^b^	2.17 ± 0.26 ^b^	2.84 ± 0.09 ^a^	2.98 ± 0.04 ^a^
Nitrogen Free Extract	2.65 ± 0.11 ^a^	1.97 ± 0.01 ^b^	1.77 ± 0.70 ^c^	1.75 ± 0.52 ^c^

Means in the same row with different superscripts differ significantly (*p* ≤ 0.05).

**Table 5:** Haematological parameters of African catfish *Clarias gariepinus* fed soaked *Jatropha curcas* kernel-based diets. Numbers are means ± standard errors.

Parameter	Soaking Time			
	0 h	24 h	48 h	72 h
Haematocrit (%)	17.33 ± 2.02 ^d^	23.16 ± 0.76 ^c^	24.83 ± 0.76 ^b^	25.5 ± 1.32 ^a^
Haemoglobin (g/dL)	5.83 ± 0.67 ^d^	7.72 ± 0.25 ^c^	8.27 ± 0.25 ^b^	8.50 ± 0.44 ^a^
Red Blood Cell (106 cells/mm^3^)	0.95 ± 0.10 ^c^	1.21 ± 0.04 ^b^	1.30 ± 0.04 ^a^	1.34 ± 0.07 ^a^
Mean Cell Volume (fL)	189.00 ± 0.41	190.41 ± 0.30	190.53 ± 0.27	190.30 ± 0.47
Mean Cell Haemoglobin (pg)	63.21 ± 0.11	63.47 ± 0.08	63.50 ± 0.07	63.43 ± 0.13
Mean Cell Haemoglobin Conc. (g/dL)	33.35 ± 0.42	33.33 ± 0.21	33.30 ± 0.36	33.33 ± 0.42

Means in the same row with different superscripts differ significantly (*p* ≤ 0.05).

## Data Availability

The data presented in this study are available on request from the corresponding authors. The data are not publicly available due to restrictions from the funding agency.
